# Genomes of Single-Stranded DNA Viruses in a Fecal Sample from South Polar Skua (Stercorarius maccormicki) on Ross Island, Antarctica

**DOI:** 10.1128/mra.00299-23

**Published:** 2023-05-18

**Authors:** Sarah R. A. Shick, Megan L. Elrod, Annie Schmidt, Simona Kraberger, David G. Ainley, Grant Ballard, Arvind Varsani

**Affiliations:** a Biodesign Center for Fundamental and Applied Microbiomics, Center for Evolution and Medicine, School of Life Sciences, Arizona State University, Tempe, Arizona, USA; b Point Blue Conservation Science, Petaluma, California, USA; c H. T. Harvey and Associates, Los Gatos, California, USA; d Structural Biology Research Unit, Department of Integrative Biomedical Sciences, University of Cape Town, Cape Town, South Africa; DOE Joint Genome Institute

## Abstract

South polar skuas migrate from subtropical regions to breed along coastal Antarctica. In a fecal sample collected on Ross Island, Antarctica, we identified 20 diverse microviruses (*Microviridae*) that share low levels of similarity to currently known microviruses; 6 appear to use a *Mycoplasma/Spiroplasma* codon translation table.

## ANNOUNCEMENT

South polar skuas (Stercorarius maccormicki) nest in coastal Antarctica and winter at sea in subtropical waters (1). Given their annual long-distance movements, these sea birds can spread pathogens between the Northern Hemisphere and the Southern Hemisphere (2). A skua fecal sample was collected off a snow patch at Cape Crozier, Ross Island, Antarctica, in December 2014. Approximately 5 g of the sample was resuspended in 20 ml of SM buffer (0.1 M NaCl, 50 mM Tris-HCl [pH 7.4], 10 mM MgSO_4_), homogenized by vortex-mixing, and centrifuged at 10,000 × *g* for 10 min. The supernatant was sequentially filtered through 0.45- and 0.2-μm (pore size) syringe filters. Viral particles in the filtrate were then precipitated with 15% (wt/vol) polyethylene glycol (PEG) 8000. The resulting solution was centrifuged at 6,000 × *g* for 20 min, and the pellet was resuspended in 2 mL of SM buffer. Of this, 200 μl was used to extract viral DNA with the High Pure viral nucleic acid kit (Roche Diagnostics, USA), and the circular DNA in this extract was enriched by rolling-circle amplification (RCA) using the TempliPhi kit (GE Healthcare). The RCA products were used to generate 170-bp insert libraries at BGI Hong Kong (using their proprietary library preparation workflow, which involved shearing with a Covaris ultrasonicator, blunting, phosphorylation, 3′-A-tailing, ligation of Illumina adapters, magnetic bead-based size fractionation, and addition of index tags by PCR) and sequenced on their Illumina 2500 sequencer. The 90-bp paired-end raw reads (131,536 paired-end reads, with an average read length of 90 nucleotides [nt]) were trimmed with Trimmomatic v0.39 (3) and *de novo* assembled with MEGAHIT v1.2.9 (4). Contigs of >1,000 nt were screened for virus-like sequences using BLASTx (5) with a RefSeq viral protein database (RefSeq release 207). All bioinformatic tools were run with default parameters, and circular viral genomes were identified based on terminal redundancy using a custom python script.

**FIG 1:**
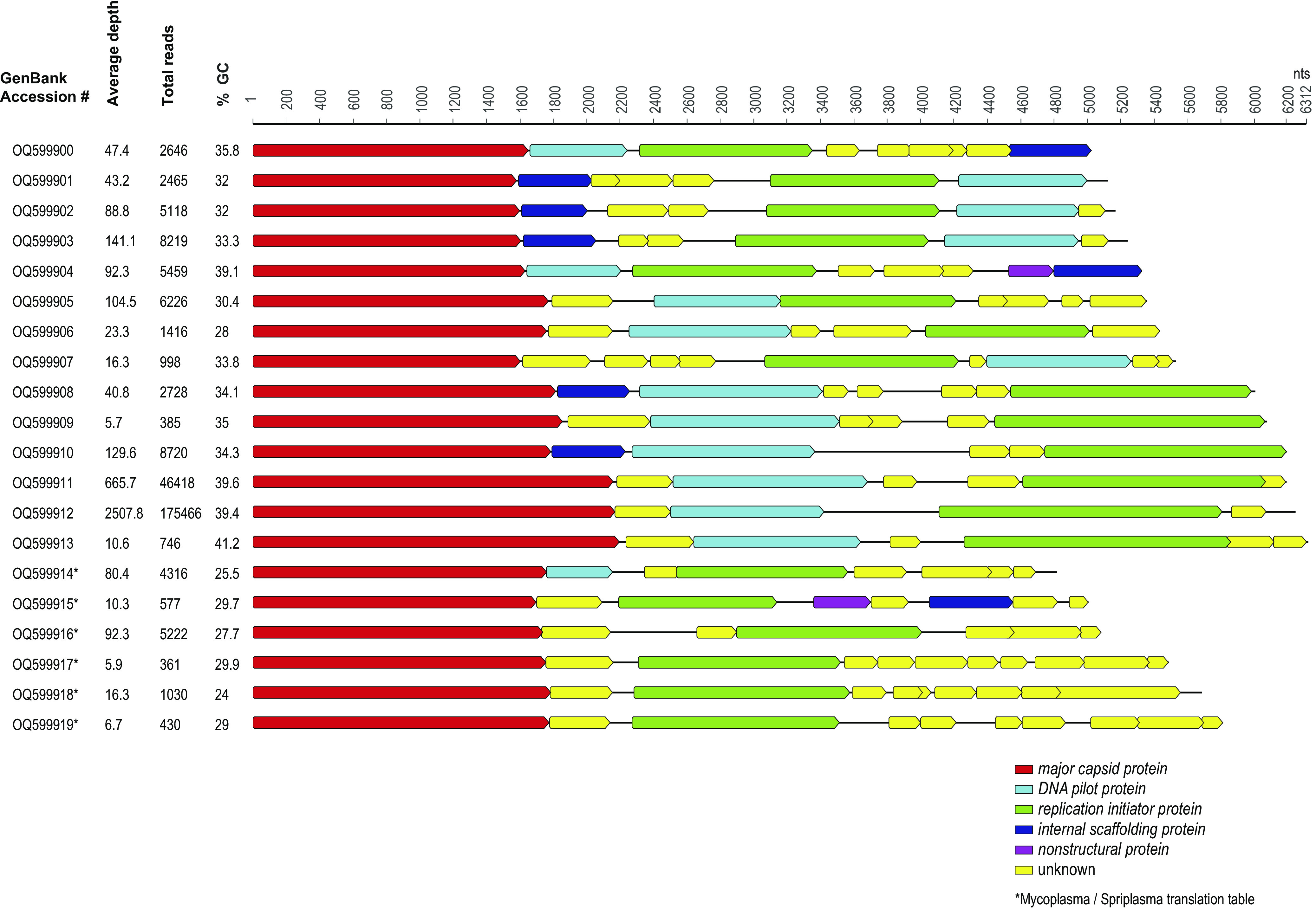
Genome organization of the 20 microviruses identified in south polar skua feces. A summary of the GC content, read depth, and number of reads mapping to each genome is provided.

We identified genomes of 20 microviruses (family *Microviridae*), which were annotated using VIBRANT (6). Microviruses are small, icosahedral, single-stranded DNA viruses that are known to infect bacteria and have been identified in various ecosystems and the feces of various animals (7–9). The 20 microviruses range in length from 4,812 to 6,312 nt, with GC contents of 24% to 41%. They have coverage depths of 5.7× to 2,507.8×, with 361 to 175,466 mapped reads (Fig. 1). All of these genomes have different genome organizations, with at least a major capsid protein (MCP) and a replication-initiator protein (Fig. 1). Six of the genomes (GenBank accession numbers OQ599914 to OQ599919) have open reading frames that use a translation table of 4 (*Mycoplasma*/*Spiroplasma*) for codon translation. *Spiroplasma*-infecting microviruses have been identified and studied previously (10–12); therefore, we are confident in the identification of the correct translation table for these 6 microviruses. None of these 6 is closely related to the only *Spiroplasma* microvirus sequence in GenBank (*Spiroplasma* virus 4 [GenBank accession number M17988]) (13). The MCP in microviruses is the most conserved protein, and BLASTp analysis revealed that the MCPs of the 20 microviruses identified here share ~28 to 51% amino acid pairwise identity; their genomes are diverse, compared to available genomes in GenBank, with genome coverage of only up to 29% for any BLASTn identity (Table 1). The 20 microviruses likely infect the enteric bacteria of south polar skuas, and they add to the diversity of microviruses that were previously identified to be associated with Antarctic animals (*n* = 51) (14) and environmental samples (*n* = 7) (15).

**TABLE 1:** Summary of the top BLASTn hits for the genomes and the top BLASTp hits for the MCPs of the 20 microviruses in south polar skua feces

Search type and GenBank accession no.	Scientific name of best hit	Strain name of best hit	Query coverage (%)	E value	Identity (%)	GenBank accession no. for best hit	Source of best-hit isolate
BLASTn							
OQ599918	*Microviridae* sp.	ctm4b9	1	2.00E−08	79.78	BK016485	Human metagenome
OQ599907	Chimpanzee feces-associated microphage 1	CPNG_29298	1	1.00E−06	77.32	KR704913	Pan troglodytes feces
OQ599906	*Microviridae* sp.	SD_HF_20	2	1.00E−05	71.53	MH572497	Ciona robusta intestinal tract
OQ599905	*Microviridae* sp.	ct9pz6	7	5.00E−29	68.75	BK047226	Human metagenome
OQ599904	Capybara microvirus Cap1_SP_164	Cap1_SP_164	7	1.00E−17	69.67	MK496737	Hydrochoerus hydrochaeris feces
OQ599903	*Microviridae* sp.	CN7_L15_514	1	5.00E−10	86.57	MT201872	Polar freshwater
OQ599902	Gokushovirus WZ-2015a	86Rcn01	29	8.00E−33	65.51	KT264834	Raccoon
OQ599901	*Microviridae* sp.	SD_HF_19	1	1.00E−12	87.50	MH572498	Ciona robusta intestinal tract
OQ599913	Sigmofec virus UA08Rod_4527	UA08Rod_4527	2	8.00E−09	71.43	OM869568	Sigmodon arizonae feces
OQ599916	*Microviridae* sp.	SD_MF_6	0	0.02	88.37	MH572485	Ciona robusta intestinal tract
OQ599900	Capybara microvirus Cap3_SP_465	Cap3_SP_465	1	2.00E−09	78.49	MK496799	Hydrochoerus hydrochaeris
OQ599915	Sigmofec virus UA08Rod_6125	UA08Rod_6125	5	5.00E−16	71.70	OM869517	Sigmodon arizonae feces
OQ599914	Gokushovirus WZ-2015a	35Fra05	2	2.00E−09	74.38	KT264783	Homo sapiens feces
OQ599912	*Microviridae* sp.	3PE-MCP-1	3	5.00E−11	71.43	MZ374777	Phrynocephalus erythrurus feces
OQ599911	Sigmofec virus UA08Rod_4138	UA08Rod_4138	11	8.00E−28	67.05	OM869575	Sigmodon arizonae feces
OQ599910	*Microvirus* sp.	1712115_239	10	2.00E−23	79.86	MT310378	Wastewater metagenome
OQ599908	*Microviridae* sp.	ctpAn10	9	9.00E−27	68.99	BK017513	Human metagenome
OQ599919	*Microviridae* sp.	ctV2r15	0	0.002	84.62	BK024956	Human metagenome
OQ599917	Chicken microvirus mg7_6	mg7_6	1	0.022	83.05	MN379637	Gallus gallus tracheal swab sample
OQ599909	*Microviridae* sp.	ctl3d8	3	5.00E−17	77.69	BK027223	Human metagenome
BLASTp							
OQ599918	*Microvirus* sp.	1712115_358	95	1.00E−64	30.16	QJB21279	Wastewater metagenome
OQ599907	Gokushovirus WZ-2015a	86Rcn01	100	2.00E−135	43.15	ALS03795	Raccoon
OQ599906	*Microvirus* sp.	gila2	96	7.00E−44	27.88	QPB07402	Heloderma suspectum
OQ599905	*Microviridae* sp.	ctEsk6	97	5.00E−103	34.85	DAT93975	Human metagenome
OQ599904	*Microvirus* sp.	1712115_459	99	6.00E−145	42.07	QJB21148	Sludge
OQ599903	*Microviridae* sp.	SD_MF_58	93	5.00E−89	35.48	AXL15386	Ciona robusta intestinal tract
OQ599902	Gokushovirus WZ-2015a	86Rcn01	96	8.00E−175	50.57	ALS03795	Raccoon
OQ599901	Gokushovirus WZ-2015a	86Rcn01	98	1.00E−139	45.73	ALS03795	Raccoon
OQ599913	Sigmofec virus UA08Rod_4686	UA08Rod_4686	100	2.00E−133	35.83	UPW41261	Sigmodon arizonae feces
OQ599916	*Microviridae* sp.	cteai15	87	6.00E−59	29.04	DAV46642	Human metagenome
OQ599900	*Microviridae* sp.	ctcj37	98	3.00E−108	35.66	AXH73167	Macaque stool
OQ599915	*Microviridae* sp.	cthYU6	97	1.00E−142	43.43	DAV99125	Human metagenome
OQ599914	*Microviridae* sp.	ctfea1	94	5.00E−93	35.48	DAW00347	Human metagenome
OQ599912	Sigmofec virus UA08Rod_4687	UA08Rod_4687	100	2.00E−148	35.80	UPW41255	Sigmodon arizonae feces
OQ599911	Sigmofec virus UA08Rod_4138	UA08Rod_4138	99	2.00E−175	44.81	UPW41319	Sigmodon arizonae feces
OQ599910	*Microviridae* sp.	ctyDb11	100	9.00E−166	43.78	DAW03743	Human metagenome
OQ599908	*Microvirus* sp.	1712115_239	100	3.00E−175	47.38	QJB21444	Wastewater metagenome
OQ599919	*Microviridae* sp.	ctfea1	93	4.00E−56	28.37	DAW00347	Human metagenome
OQ599917	Capybara microvirus Cap3_SP_562	Cap3_SP_562	89	1.00E−60	30.68	QCS36647	Hydrochoerus hydrochaeris feces
OQ599909	*Microviridae* sp.	ct7vS1	100	8.00E−128	38.12	DAI40795	Human metagenome

### Data availability.

The microvirus sequences have been deposited in NCBI databases under BioProject accession number PRJNA874327, BioSample accession number SAMN33378344, SRA accession number SRR23587964, and GenBank accession numbers OQ599900 to OQ599919.
